# LGL leukemia patients exhibit substantial protective humoral responses following SARS‐CoV‐2 vaccination

**DOI:** 10.1002/jha2.472

**Published:** 2022-05-31

**Authors:** Heejin Cheon, Omar Elghawy, Bryna C. Shemo, David J. Feith, Thomas P. Loughran

**Affiliations:** ^1^ University of Virginia Cancer Center Charlottesville Virginia USA

**Keywords:** blood diseases, haematological malignancies, leukaemia, lymphoid malignancies, virology

## Abstract

Large granular lymphocyte leukemia is a rare chronic lymphoproliferative disorder of cytotoxic cells. Other hematological malignancies such as CLL and multiple myeloma have been associated with poor vaccination response and markedly increased severe acute respiratory syndrome coronavirus 2 (SARS‐CoV‐2) mortality rates, specifically in patients who have undergone immunosuppressive therapy. Given the immunosuppressive therapies often used to treat the disease, large granular lymphocytic (LGL) patients may be especially vulnerable to SARS‐CoV‐2 infection. A questionnaire was sent to all patients in the LGL Leukemia Registry at the University of Virginia (UVA) to obtain information on vaccination status, type of vaccine received, side effects of vaccination, patient treatment status before, during, and after vaccination, antibody testing, history of coronavirus disease 2019 (COVID‐19) infection, and presence or absence of booster vaccination. Antibody testing of 27 patients who had quantitative SARS‐CoV‐2 Spike Protein IgG levels determined by University of Virginia medical laboratories via the Abbott Architect SARS‐CoV‐2 IgG II assay were collected. The assay was scored as reactive at a threshold of ≥50.0 AU/mL or nonreactive with a threshold of <50.0 AU/mL. LGL patients without treatment as well as patients who held treatment prior to their vaccination have a robust humoral response to SARS‐CoV‐2 vaccines. Patients who did not hold their immunosuppressive treatments have signifigantly diminished vaccine response compared to those who held their immunosuppressive treatment. Our findings support a dual strategy of pausing immunotherapy during the vaccination window and administration of the SARS‐CoV‐2 booster to all LGL leukemia patients to maximize protective antibodies.

## 1 INTRODUCTION

Large granular lymphocyte (LGL) leukemia is a rare chronic lymphoproliferative disorder of cytotoxic effector cells, namely CD8+ T and/or natural killer (NK) cells [1]. It typically affects elderly individuals with a median onset of age 60 [2]. While some patients face an indolent course of disease not necessitating treatment, the majority of patients are symptomatic at presentation [1]. Usual treatment regimens include single agent immunosuppressives such as methotrexate, cyclophosphamide, and cyclosporine [1]. By nature of the immunosuppressive therapies and epidemiologic parameters, LGL leukemia patients may be especially vulnerable to severe acute respiratory syndrome coronavirus 2 (SARS‐CoV‐2) infection. Similar hematologic malignancies like chronic lymphocytic leukemia (CLL) have been associated with markedly increased SARS‐CoV‐2 mortality rates, specifically in patients who have undergone immunosuppressive therapy [3]. Weaker vaccination responses were also observed in a cohort of multiple myeloma patients [4]. Given the previously reported coexistence of T cell‐large granular lymphocytic leukemia (T‐LGL) leukemia with B cell dyscrasias, one must wonder whether insufficient humoral responses to SARS‐CoV‐2 vaccination would be observed among LGL patients [5, 6, 7, 8, 9].

About 30% of LGL leukemia patients have a concurrent autoimmune disorder, most commonly rheumatoid arthritis [1]. Notably, the therapeutic approach for LGL leukemia is often similar in autoimmune diseases. In one study of SARS‐CoV‐2 vaccine humoral responses on a cohort receiving methotrexate for psoriasis, patients were found to have detectable antibody titers even with concomitant immunosuppressive therapy [10]. Moreover, patients receiving methotrexate for rheumatoid arthritis demonstrated equivalent response rates to the SARS‐CoV‐2 vaccine versus healthy controls [11]. In both of these studies, antibody titers were specifically measured as they are directly correlated with protective immunity from SARS‐CoV‐2 [12]. Currently, there are no reports examining the intrinsic SARS‐CoV‐2 vaccine responses in LGL leukemia. Therefore, we examined vaccine titer responses in LGL leukemia patients to determine if LGL leukemia patients develop robust humoral immune response with or without concurrent immunosuppressive treatment.

## 2 METHODS

A questionnaire was sent to all patients in the large granular lymphocytic (LGL) Leukemia Registry at the University of Virginia (UVA) to obtain information on vaccination status, type of vaccine received, side effects of vaccination, patient treatment status before, during, and after vaccination, antibody testing, history of coronavirus disease 2019 (COVID‐19) infection, and presence or absence of booster vaccination. To identify additional patients with antibody testing, the EPIC electronic medical records of patients seen in the LGL leukemia clinic at the UVA were reviewed. Demographic information, vaccination status, patient treatment status before, during, and after vaccination, history of COVID‐19 infection, and presence or absence of booster vaccination were collected. Table 1 summarizes the clinical features of our cohort.

**TABLE 1:** Summary of clinical characteristics of LGL patient cohort

	UVA cohort	Expanded cohort
	*n* = 27	*n* = 68
Age (median)	58 (23–75)	60 (23–82)
Sex		
Male	37.0% (10/27)	39.7% (27/68)
Female	63.0% (17/27)	60.3% (41/68)
LGL leukemia type		
T cell‐large granular lymphocytic leukemia	100% (27/27)	97.1% (66/68)
Natural killer (NK)‐LGL	0% (0/27)	2.9% (2/68)
SARS‐CoV‐2 vaccine type		
Astra Zeneca	0% (0/25)	3.0% (2/66)
Johnson and Johnson	4.0% (1/25)	3.0% (2/66)
Moderna	32.0% (8/25)	27.2% (18/66)
Pfizer	44.0% (11/25)	51.5% (34/66)
Unknown	20.0% (5/25)	15.2% (10/66)
Treatments		
Methotrexate	40.7% (11/27)	32.4% (22/68)
Cyclophosphamide	0% (0/27)	1.5% (1/68)
Cyclosporine	3.7% (1/27)	11.8% (8/68)
Other*	7.4% (2/27)	5.9% (4/68)
None	48.1% (13/27)	47.1% (32/68)
Unknown**	0% (0/27)	1.5% (1/68)
Treatment pause status***		
Yes	42.9% (6/14)	36.1% (13/36)
No	50.0% (7/14)	55.6% (20/36)
Unknown	7.1% (1/14)	8.3% (3/36)
Vaccine booster status (before titer)		
Yes	22.2% (6/27)	13.2% (9/68)
No	77.8% (21/27)	86.8% (59/68)
Other medical conditions (*n* = 59)	Responded to vaccine	Not responded to vaccine
Autoimmune^†^	61.5% (8/13)	35.7% (5/13)
Cardiac^‡^	0% (0/3)	100% (3/3)
Malignancy^⁺^	100% (7/7)	0% (0/7)

* Other treatments include etanercept, adalimumab, hydroxychloroquine, and filgrastim.

** Data are unavailable.

*** Patients who were never initiated on treatment were excluded.

^†^ Rheumatoid arthritis, Sjogren's, lupus, celiac, sarcoidosis, Grave's disease, psoriatic arthritis.

^‡^ Valvular heart disease, coronary artery disease, myocardial infraction.

^⁺^ Melanoma, squamous cell carcinoma, myeloma, papillary thyroid cancer, ductal carcinoma.

Antibody testing included 27 patients who had quantitative SARS‐CoV‐2 Spike Protein IgG levels determined by UVA medical laboratories via the Abbott Architect SARS‐CoV‐2 IgG II assay. The assay was scored as reactive at a threshold of ≥50.0 AU/ml or nonreactive with a threshold of <50.0 AU/ml. Patients with alternative quantitative antibody assays were not included in the quantitative analysis of antibody titers, although they were noted qualitatively as reactive or nonreactive and downstream analysis was noted. Instances where the target of the antibody test could not be confirmed (nucleocapsid or spike protein) were excluded from antibody response entirely. Patients with known incidence of SARS‐CoV‐2 infection were also excluded.

Statistical analysis was conducted using R software. Welch two sample *t*‐test and Fisher's exact test were utilized where appropriate.

## 3 RESULTS AND DISCUSSION

**FIGURE 1:**
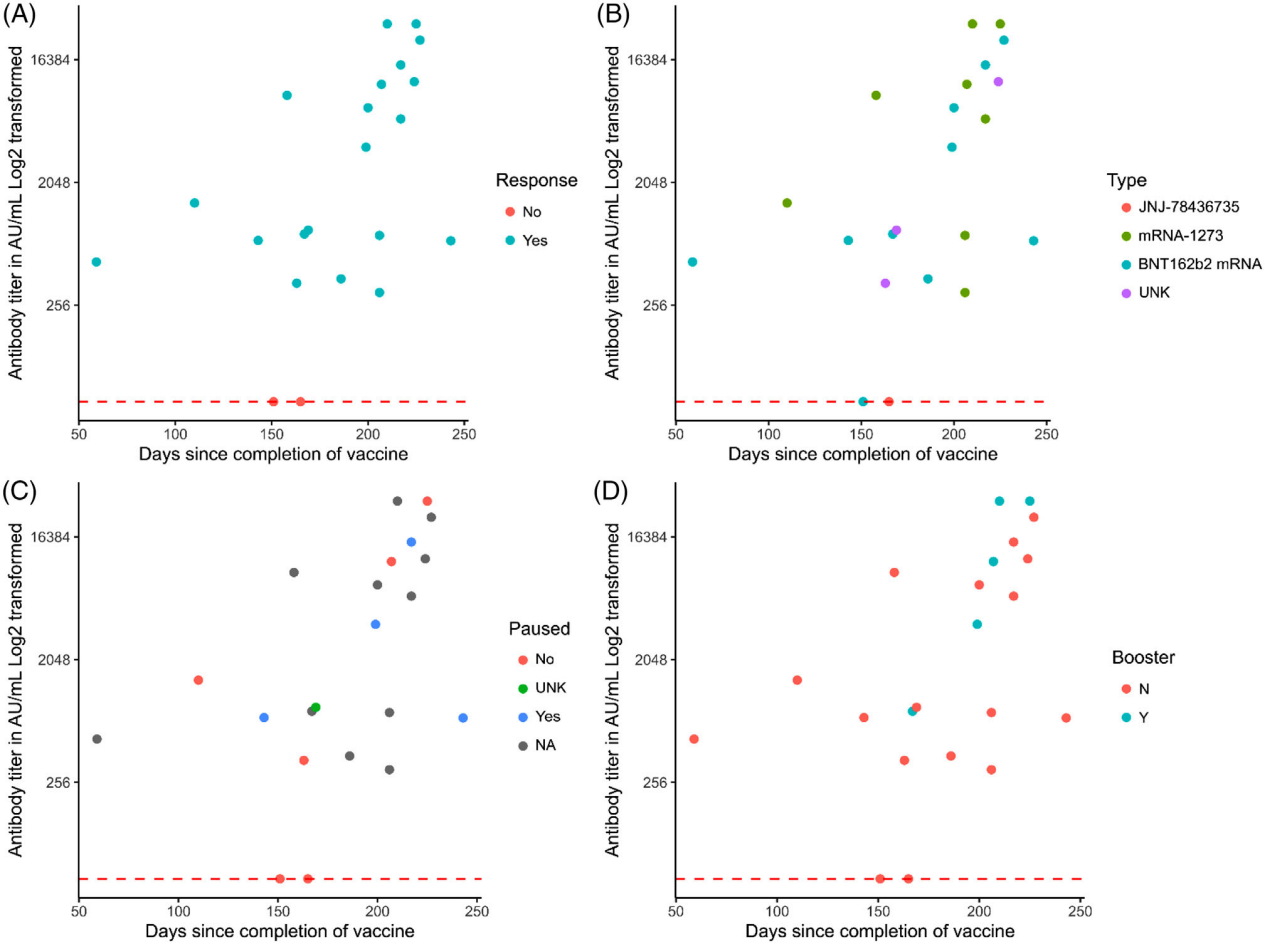
T‐LGL patient SARS‐CoV‐2 antibody titers. (A–D) Plots of patients in the UVA cohort within the full cohort with available data on number of days since completion of the first series of SARS‐CoV‐2 vaccination. Antibody response titer is plotted against days since completion of vaccine, with a dashed line at 50 indicating antibody titer threshold for response to vaccine. Data on expanded cohort are listed in Table 1. (A) Response to vaccine, as determined by the presence of antibody titer greater than 50 AU/ml, is indicated by color annotation. (B) Type of SARS‐CoV‐2 vaccine received is indicated by color annotation. (C) Immunosuppressive medication pause status is indicated by color annotation. (D) SARS‐CoV‐2 booster status is indicated by color annotation if data are available. UNK = Unknown. NA = Patient is not initiated on LGL treatment

The qualitative vaccine response rate was 83.8% (57/68) in the LGL registry's full cohort (*n* = 68). This included a subset of UVA patients (*n* = 27) with quantitative antibody titers, and 41 outside patients whose antibody titers were collected at other sites. In the full cohort, there were no known previous COVID‐19 infections and nine total patients received booster shots. When patients with booster shots were excluded, the response rate was 81.3% (48/59) in the full cohort and 85.7% (18/21) in the UVA cohort for those who had available qualitative data on response rate (Table 1). Among the UVA cohort, we observed SARS‐CoV‐2 vaccine responses in 88.9% (24/27) patients including six patients who received a booster dose, verified through quantitative SARS‐CoV‐2 Spike Protein IgG (Figure 1A).

Antibody responses to mRNA‐1273, BNT162b2 mRNA, and JNJ‐78436735 vaccines are plotted in Figure 1B. There were no statistically significant differences between antibody titers elicited by mRNA‐1273 or BNT162b2 mRNA vaccines (*p* = 0.2261; Welch two sample *t*‐test). One patient received JNJ‐78436735 vaccine in the UVA cohort and reported a titer of <50 AU/ml, indicating no response.

Seven patients with quantitative antibody data had continued their methotrexate or adalimumab treatments with vaccination, while six patients had paused methotrexate treatment before, during, and/or after vaccination (Figure 1C). Time for stopping therapy before and after vaccination ranged from 10 days before the first vaccine to 10 days after the second vaccine. Given the variability of vaccine responses in patients with LGL leukemia as seen in Figure 1A, we asked if withholding immunosuppressive medication before, during, or after vaccination improved humoral responses to the vaccine (*n* = 28). This analysis excluded those who received booster vaccines before the quantitative antibody draw and those with known COVID‐19 infection. We observed that LGL patients who withheld their medications were more likely to respond to the vaccine than those who continued their medication (*p *= 0.0039; Fisher's exact test). Notably, all patients who paused their medication had a detectable titer, indicating response to the vaccine. Finally, patients who received a booster dose trended toward having a higher antibody titer in the full cohort (Figure 1D; *n* = 27; *p *= 0.1054; Welch two sample *t*‐test).

Next, we gathered autoimmune, cardiac, and malignancy data on 59 patients listed on this study and examined their association with response status to COVID19 vaccination. We have excluded those with prior COVID19 infection and those who received a booster vaccine. We have observed that those with autoimmune conditions (rheumatoid arthritis, Sjogren's, lupus, celiac, sarcoidosis, grave's disease, psoriatic arthritis) had a tendency toward having a lower probability of responding to COVID19 vaccination compared to those without autoimmune disorders, although this finding was not statistically significant (*p* = 0.053, Fisher's Exact Test). Notably, we have observed that all three patients with cardiac conditions (valvular heart disease, coronary artery disease, myocardial infraction) did not respond to COVID19 vaccination (*p* = 0.005, Fisher's Exact Test). Nonetheless, this is a small number of patients with cardiac co‐comorbidities, and drawing significant conclusions is limited with our current sample size. Lastly, no association with concurrent or previous history of malignancies (melanoma, squamous cell carcinoma, myeloma, papillary thyroid cancer, ductal carcinoma) was found for COVID19 vaccine response (*p* = 0.328, Fisher's Exact Test) with all seven patients with malignancy responding to the vaccine.

We observed that LGL patients in general have a robust humoral response to SARS‐CoV‐2 vaccines unlike other hematologic malignancies, such as CLL. Among LGL leukemia treatment modalities, methotrexate is one of the first‐line immunotherapies, but concomitant use has been previously shown to weaken response to the SARS‐CoV‐2 vaccine [13, 14]. Our results confirm these findings to hold true in LGL leukemia. The patient subset that paused methotrexate treatment within the vaccination window demonstrated better response to the vaccine. Additionally, patients who received the booster were documented to have higher antibody titers thus providing another intervention to protect at‐risk patients, with the qualifier that their titers were taken soon after their booster shot. Given the poor prognosis of SARS‐CoV‐2 in patients with hematologic malignancies, it is critical that proper measures are taken to ensure the efficacy of vaccination in these populations [15]. Our findings support a dual strategy of pausing immunotherapy during the vaccination window and administration of the SARS‐CoV‐2 booster to all eligible LGL leukemia patients to maximize protective antibody response.

## CONFLICT OF INTEREST

Thomas P. Loughran Jr. is on the Scientific Advisory Board and has stock options for Keystone Nano, Bioniz Therapeutics, and Dren Bio. Thomas P. Loughran Jr. and David Feith received honoraria from Kymera Therapeutics. David J. Feith has research funding from AstraZeneca. Other authors have no conflict of interest with the work presented in this manuscript.

## ETHICS STATEMENT

Data collection and analysis were performed in concordance with the University of Virginia Institutional Review Board under the protocols IRB HSR 17070 and 17000. This research adhered to the tenets of the Declaration of Helsinki and was conducted in accordance with regulations set forth by the Health Insurance Portability and Accountability Act.

## AUTHOR CONTRIBUTIONS

Thomas P. Loughran Jr., David J. Feith, Bryna C. Shemo, and Heejin Cheon designed the study. Omar Elghawy and Bryna C. Shemo compiled the data. Heejin Cheon performed the analysis and generated the figures. Heejin Cheon and Omar Elghawy wrote the paper with contributions from all co‐authors.

## Data Availability

The data that support the findings of this study are available on request from the corresponding author. The data are not publicly available due to privacy or ethical restrictions.
